# Supplementary material for: Restoration of normal central pain processing following manual therapy in nonspecific chronic neck pain
Source: PLoS One. 2024 May 23;19(5):e0294100. doi: 10.1371/journal.pone.0294100 (PMC11115211; doi:10.1371/journal.pone.0294100)
Supplement: S1 File — (PDF) [file pone.0294100.s003.pdf]

M10 - Memoria para el CEISH: proyecto de investigación con seres humanos

NoRefCEID: M10/2018/160

Datos del/la solicitante

Nombre: JOSU ZABALA MATA

DNI/NIF: [redacted]

Telefono: [redacted]

E-mail: [redacted]

Campus: [redacted]

Centro: [redacted]

Departamento: [redacted]

Área: [redacted]

TÍTULO DEL PROYECTO

Introducir el título identificativo del proyecto.

Este título debe ser el mismo que aparezca en las memorias así como en todos los documentos que las acompañan (hojas de información y consentimiento, autorizaciones, solicitudes a diputaciones etc)

Relación entre el estado pro-anti nociceptivo y el estado clínico de los pacientes con dolor de cuello, antes y después de un tratamiento de fisioterapia.

TIPO DE ACTIVIDAD

Proyecto de investigación

FINANCIACIÓN

¿Se dispone de financiación para la realización del proyecto?

No

Indicar la fuente de financiación:

Estado de la financiación:

No procede

FECHAS CLAVE DEL PROYECTO

Fecha prevista inicio del proyecto

03/12/2018

Fecha prevista fin del proyecto

26/06/2020

RESUMEN DEL PROYECTO

Sintetizar (no más de 250 palabras) los aspectos más importantes del trabajo: objetivos, hipótesis y variables del estudio. Teniendo siempre en cuenta el comité o los comités a los que va dirigido y la información que les pueda ser útil y necesaria para realizar la evaluación.

El proyecto incluye 2 estudios: un estudio experimental que valorará la relación entre el estado pro-anti nociceptivo y el estado clínico de pacientes con dolor cervical crónico (DCC), antes y después de un tratamiento de fisioterapia. Y segundo, un estudio caso-control que aprovechará los datos adquiridos en la primera sesión del estudio experimental para compararlos con sujetos sanos.

60 sujetos con DCC y otros 20 sujetos sanos serán evaluados. Se realizarán por un lado mediciones relacionados con el procesamiento del dolor que consistirán en tres valores; modulación condicionada del dolor (CPM), suma temporal del dolor (TSP) y umbral del dolor a la presión (PPT). Por otro lado se valorarán las características clínicas que consistirán en diferentes cuestionarios; Neck Dissability Index (NDI), Pain Catastrophization Scale (PCS), Pain Relationed Function (PRF) y Numerical Rating Scale (NRS). A continuación se les aplicará un tratamiento convencional de fisioterapia de 4 semanas de duración máxima, realizando una sesión por semana. El estudio se realizará en la consulta Hiru Fisioterapia SCP del fisioterapeuta Josu Zabala Mata (calle Antso estegiz, 4. Durango).

Las mediciones se realizarán el primer día, al finalizar el tratamiento y a un año vista. Esto nos dará la posibilidad de relacionar los valores de PPT, CPM, y TSP con las variaciones en el estado clínico de los pacientes. Esto nos puede ayudar a determinar qué factores podrían ser predictivos de la efectividad de un tratamiento.

Asimismo se reclutarán sujetos control (asintomáticos), a quienes solamente se les realizarán las valoraciones de PPT, CPM y TSP. El objetivo será comparar diferencias en el estado del procesamiento del dolor entre sujetos con dolor cervical crónico y asintomáticos.

Memorias del proyecto

Comité	NoRefCEID	Nombre	Estado
CEISH	M10/2018/160	0	Completada

Parte I: Valor social y justificación

1.1 VALOR SOCIAL DEL PROYECTO

A continuación se muestra la finalidad principal del proyecto o campo en el que se espera un aumento del conocimiento o un beneficio social último que justifique su realización.

2. Investigación aplicada a la prevención, diagnóstico o tratamiento de alteraciones o de enfermedades físicas, psíquicas u otras anomalías o de sus efectos en los seres humanos, los animales o las plantas.

1.2 JUSTIFICACIÓN DEL USO DE SERES HUMANOS (BENEFICIOS)

Exponer brevemente los beneficios que se esperan obtener para los sujetos seleccionados para la muestra.

Dado que el estudio va a investigar los procesamiento del dolor en sujetos con dolor cervical, la utilización de seres humanos como sujetos de estudio es indispensable.

Parte II: Cualificación equipo investigador

2.1 PERSONAL QUE LLEVARÁ A CABO LAS TAREAS CON SERES HUMANOS, SUS MUESTRAS Y/O SUS DATOS

A continuación se muestran todas las personas integrantes del equipo investigador includo el/la solicitante. Añadir en este apartado cualquier otra persona que colabore en el proyecto como reclutadores, encuestadores etc. En el caso de proyecto tutelado añadir al tutor o director del mismo.

- Datos identificativos (nombre, NIF, vinculación y nivel académico)
- Tareas concretas que realizará de acuerdo a lo descrito en el apartado '3.2 Diseño metodológico'(añadir cuantas tareas sean necesarias)
- Formación y experiencia previa en cada tarea en particular (realización de encuestas, reclutamiento, toma de muestras, tratamiento de datos, trabajo en laboratorio, dirección de tesis etc)

1. ION LASKURAIN AGIRREBEÑA

NIF: [redacted]

Vinculación: [redacted]

Nivel académico: Ldo./Graduado

Tarea: Diseño y supervisión del proyecto

Experiencia: Licenciado en Fisioterapia, Master en Fisioterapia Deportiva, Master en Fisioterapia avanzada y actualmente Doctorando en King´s College London (Londres, Reino Unido). A formado parte de diversas investigaciones, y tiene una amplia experiencia en el tratamiento de pacientes con cervicalgia.

2. JON JATSU AZKUE BARRENETXEA

NIF: [redacted]

Vinculación: [redacted]

Nivel académico: Doctor

Tarea: Diseño y supervisión del proyecto

Experiencia: - Doctor en Medicina y Cirugía, Universidad del País Vasco. Máster en TIC y entornos virtuales de formación, Grupo 9 Universidades - Profesor Titular de Universidad, Facultad de Medicina Universidad País Vasco, 2009- - Investigador Predoctoral, Dpto Neurociencias Universidad del País Vasco, 1992-1995 - Investigador Postdoctoral, II Physiologisches Institut Universität Heidelberg (Alemania), 1995-1997 - Investigador Postdoctoral, Departamento Neurociencias Universidad del País Vasco, 1998-2001 - Investigador del Programa Ramón y Cajal del Minist

3. JOSU ZABALA MATA

NIF: [redacted]

Vinculación: [redacted]

Nivel académico: Ldo./Graduado

Tarea: Diseño, reclutamiento y medición de variables

Experiencia: Licenciado en Ciencias de la Actividad Física y del Deporte, y Graduado en Fisioterapia. Máster en Investigación Translacional en Fisioterapia. Actualmente profesor asociado en EHU/UPV. Más de 10 años de experiencia en terapia manual, con una amplia experiencia en el tratamiento de pacientes con dolor crónico.

4. **MIKEL LARRAÑAGA ETXABURU**

   NIF: [redacted]

   Vinculación: Externo/a

   Nivel académico: Ldo./Graduado

   Tarea: Intervención

   Experiencia: Graduada en fisioterapia (nº col.1647).Más de 10 años de experiencia en terapia manual

5. **ARAITZ IRAZU LEGORBURU**

   NIF: [redacted]

   Vinculación: Externo/a

   Nivel académico: Ldo./Graduado

   Tarea: Intervención

   Experiencia: Graduada en fisioterapia (nº col.1648). Más de 10 años de experiencia en terapia manual

Parte III: Validez científica y metodológica

3.1 OBJETIVOS CIENTÍFICOS DEL PROYECTO

Describir brevemente los principales objetivos que se pretenden alcanzar con este proyecto.

1. Investigar la relación entre el estado pro-antinociceptivos y el estado clínico de los pacientes con dolor cervical crónico.

2. Investigar la relación de las variaciones del estado pro-antinociceptivos y el estado clínico de los pacientes con dolor cervical crónico tras un tratamiento de fisioterapia, y a un año vista.

3. Investigar las diferencias en el estado pro-antinociceptivos entre pacientes con dolor cervical crónico y grupo control.

3.2 DISEÑO METODOLÓGICO DEL PROYECTO

Describir a continuación

- El **tipo de estudio** (cualitativo, cuantitativo, prospectivo, observacional, caso-control, doble ciego etc.)
- La **muestra**: tamaño, justificación, características, criterios de inclusión/exclusión
- Las **variables** de estudio
- El **modelo de análisis de resultados previsto**

Tener en cuenta el comité o los comités a los que va dirigido y la información que les pueda ser útil y necesaria para realizar la evaluación.

1.Tipo de estudio:

Se va a realizar un estudio experimental para medir la relación entre los parámetros clínicos y el procesamiento del dolor en sujetos con dolor de cuello tras un tratamiento de fisioterapia. Asimismo, se compararán con sujetos control (asintomáticos) las mediciones realizadas en la primera visita (caso-control).

2.Lugar, reclutamiento y tamaño muestral:

El estudio se llevará a cabo en 2 localizaciones:

- Consulta del Fisioterapeuta Josu Zabala Mata (Antso Estegiz 4, Durango).

- Laboratorio del dolor del departamento de neurociencias de la UPV (Leioa).

El reclutamiento lo realizará Josu Zabala Mata. El grupo de intervención se reclutará en el centro Hiru Fisioterapia SCP, donde todos los pacientes que acudan y cumplan con los criterios de inclusión serán propuestos para tomar parte en el estudio. El grupo control será reclutado en la universidad mediante un poster informativo que se pondrá en la puerta del despacho de Josu Zabala Mata.

Con una significación estadística de 0.05 y una potencia de 90%, se requerirán 60 sujetos para detectar una relación entre factores de procesamiento del dolor y la mejora sintomática, donde aquellos con alteración del procesamiento del dolor se puedan clasificar como malos respondedores a un tratamiento (odds ratio 3.45)

3. Criterios de inclusión y exclusión:

Los criterios de inclusión para sujetos con dolor de cuello y sujetos control:

- Mayores de 18 años

Además, los sujetos con dolor de cuello presentarán:

- Dolor de cuello intermitente

- Que se agrava o mejora con determinados movimientos del cuello o determinadas posturas

- Que no haya sido causado por un golpe, caída o accidente

Criterios de exclusión para sujetos sintomáticos:

- Presenten una cervicobraquialgia

- Hayan sido o estén esperando una intervención quirúrgica del cuello

- Hayan sido diagnosticadas de una enfermedad inflamatoria o patología específica de la columna (por ejemplo artritis reumatoide)

- En cuyo tratamiento las movilizaciones cervicales y de los tejidos blandos estén contraindicadas

Criterios de exclusión para sujetos control:

- Presencia de dolor intermitente de más de 3 meses de duración en cualquier parte del cuerpo

- Patologías sistémicas, cardíacas o neurológicas que cursen con alteración de la sensibilidad

-Toma de medicación que altera la capacidad sensitiva

4. Intervención (sólo para sujetos con dolor de cuello)

Se realizará un tratamiento de fisioterapia con una duración máxima de 4 semanas, aplicando una intervención semanal de alrededor de 45 minutos. El tratamiento consistirá en tres vías de trabajo; por un lado se trabajará el componente miofascial, por otro el componente articular y por último el control motor. Para el componente miofascial se realizarán técnicas fasciales y tratamiento de los puntos gatillos miofasciales (PGM). El tratamiento articular consistirá en movimientos pasivos a baja velocidad, incluyendo translaciones segmentarios, deslizamientos accesorios y movimientos fisiológicos tanto durante como al final del recorrido articular. El control motor se trabajará en consulta y se mandarán ejercicios específicos para casa. La realización de las diferentes técnicas dependerá de la valoración fisioterapéutica realizada el primer día y de la evolución del tratamiento. Dicha valoración será realizada por el investigador principal y el tratamiento lo realizarán 2 fisioterapeutas con una experiencia en terapia manual de más de 10 años.

El tratamiento propuesto es el que se realiza de forma habitual en este centro de fisioterapia.

5. Variables descriptivas del sujeto

- Edad, altura, peso

- Fecha de aparición de los síntomas

- Localización del dolor

- Intensidad media del dolor cervical en las últimas 24 horas, utilizando una escala visual analógica (Angmo et al. , 2016) y presencia de dolor durante los movimientos del cuello

- Discapacidad producida por la cervicalgia, utilizando el Índice de Discapacidad Cervical (Neck

Disability Index) validado al castellano (Andrade Ortega et al., 2010)

- Percepción catastrófica de la lesión, utilizando la escala de catastrofismo del dolor (Pain Catastrophizing Scale) validado al castellano (Campayo et al., 2008)

6. Variables e instrumentos de medición relativas a los parámetros del procesamiento del dolor

6.1. Umbral de dolor a la presión (PPT): las medidas de UDP se realizarán con un algómetro digital de presión FISHER con el punto de apoyo de 1 cm de diámetro (Pain Diagnostics and Thermography Inc, Great Neck, NY), tanto en el grupo sano como en el afecto. El UDP se define como la mínima presión con la que la sensación de presión se convierte en sensación dolorosa (Fisher et al., 1990). El UDP se aplicará en dos localizaciones diferentes y de forma bilateral. El primero será en el ángulo de las fibras superiores del trapecio, entre 5 y 8 cm superior y medial al ángulo superior de la escápula. El segundo punto será en el tibial anterior, 2.5 cm lateral y 5 cm inferior respecto a la tuberosidad tibial anterior. Los sujetos serán instruidos para que informen al investigador de la primera sensación de dolor percibida. La presión se realizará a un Kg por segundo hasta la señal del sujeto, momento donde se registrará el valor conseguido. Se aplicarán tres mediciones en cada localización, con un intervalo de 1 minuto, y se utilizará la media de los tres valores para el estudio. Este procedimiento mostró un alto coeficiente de correlación intraclase (Chesterton et al., 2007).

6.2. Sumación temporal del dolor (TSP)

6.2.1. Búsqueda del umbral del dolor: Las mediciones se realizarán en un ambiente tranquilo, con los sujetos sentados enfrente de una mesa donde apoyarán su mano. Dos electrodos autoadhesivos se colocarán en el dorso de la mano dominante, a dos cm de separación una de la otra. Se aplicará un tren de 5 impulsos con 1 ms de duración y 4ms de diferencia. En total 21 ms. Para ello utilizaremos un electroestimulador DS7, la cual tras calcular la impedancia de la piel donde se aplican los impulsos, modifica la potencia para que la intensidad de estímulo que le llega al individuo sea de los mA deseados. El electroestimulador estará a su vez conectado al ordenador, el cual mediante un programa específico, posibilitará la aplicación de la frecuencia deseada. Se aplicará el tren de forma escalonada, subiendo la intensidad hasta conseguir el umbral del dolor. Dicha subida se realizará de 0.2 en 0.2 mA. A continuación, empezando por un mA por encima del umbral delimitado en la subida, iremos bajando a la misma frecuencia hasta que el estímulo se note como no doloroso. Este procedimiento se realizará dos veces y cogeremos la media de los 4 valores obtenidos.

6.2.2. Búsqueda del Wind Up: Protocolo; Aplicación de un tren de 5 trenes (5 impulsos en cada tren) con una frecuencia de 3 Hz y la intensidad de 1.2 respecto umbral. Primero se evaluará el dolor del primer impulso del primer tren mediante la escala verbal numérica de clasificación del dolor. Tras 3 minutos de descanso, se volverá a aplicar el mismo tren y se le pedirá que cuantifique la intensidad de dolor del último impulso. Por último sacaremos un ratio de la diferencia de dolor percibido.

Este procedimiento no supone ningún sufrimiento para los sujetos ya que las aplicaciones del estímulo eléctrico están muy cerca del umbral del dolor.

6.3. Modulación condicionada al dolor (CPM): Realizaremos una medición del PPT con el algómetro digital (anteriormente citado) en el primer espacio intermetatarsal de la mano homolateral al dolor de cuello, o en caso de dolor bilateral, en la mano dominante. A continuación introduciremos el pie contralateral a la mano utilizada en un cubo de agua con hielos. Mantendremos el pie inmerso durante 2 minutos. En el caso que el sujeto no tolerase dicho tiempo se retirará el pie del agua.

Volveremos a realizar las mediciones del umbral del dolor mediante algómetro tanto durante (pasados 30 segundos) como inmediatamente después de sacar el pie del agua. Utilizaremos los valores obtenidos de los umbrales del dolor antes, durante y después para medir el efecto de las vías inhibitorias del dolor. Esta metodología ha mostrado buena a excelente fiabilidad intrasesión (Lewis et al., 2012; Kennedy et al., 2016).

7. Variables de medición relativas a los efectos de las intervenciones

7.1. Escala de impresión de mejoría global del paciente (PGI-I: Patient Global Impression of Improvement Scale): Se utilizará el PGI-I para medir la respuesta sintomática del participante a la intervención (Farrar et al., 2001). El PGI-I consiste en una sola pregunta que solicita al participante que clasifique el alivio obtenido con la intervención. El participante escoge de una escala likert de 7 puntos (Muchísimo mejor; Mucho mejor; Un poco mejor; Ningún cambio; Un poco peor; Mucho peor; Muchísimo peor) la respuesta que más se ajuste al efecto. Se utilizará la version española desarrollada por la Sociedad Española del Dolor.

8. Personas que realizarán la intervención y mediciones:

Todas las mediciones se realizarán por la misma persona (Josu Zabala Mata) para asegurar una adecuada estandarización de las mismas. Las mediciones tendrán una duración aproximada de 1 hora. El estudio con los pacientes se realizará en la consulta Hiru Fisioterapia SCP, del Fisioterapeuta Josu Zabala Mata. Las intervenciones las realizarán los fisioterapeutas Araitz Irazu Legorburu y Mikel Larrañaga Etxaburu. Las mediciones a los sujetos control se realizarán en el laboratorio de dolor del departamento de neurociencias.

9. Análisis.

Se realizarán 3 tipos de análisis:

1. Comparación de las variables del procesamiento del dolor entre sujetos con dolor de cuello y control.

2. Comparación de las variables clínicas y del procesamiento del dolor en los sujetos con dolor de cuello

3. Análisis de las variables clínicas y del procesamiento del dolor tras el tratamiento de fisioterapia.

REFERENCIAS

Angmo P, Mohanty P, Pattnaik M. Effects of unilateral posteroanterior mobilization in subjects with sacralized lumbosacral transitional vertebrae. Journal of bodywork and movement therapies.2016;20:19-25.

Andrade Ortega JA, Delgado Martinez AD, Almecija Ruiz R. Validation of the Spanish version of the Neck Disability Index. Spine.2010;35:E114-E8.

Campayo JG, Rodero B, Alda M. Validation of the Spanish version of the Pain Catastrophizing Scale in fibromialgia. Medicina Clínica.2008; 131:487-93.

Chesterton LS, Sim J, Wright CC, Foster NE. Interrater reliability of algometry in measuring pressure pain thresholds in healthy humans, using multiple raters. Clin J Pain. 2007;23(9):760-766.

Lewis GN, Heals L, Rice DA, Rome K, McNair PJ. Reliability of the conditioned pain modulation paradigm to assess endogenous inhibitory pain pathways. Pain Res Manag 2012;17:98-102.

Kennedy DL, Kemp HI, Ridout D, Yarnitsky D, Rice AS. Reliability of conditioned pain modulation. PAIN 2016;157:2410-19.

Farrar JT, Young JP, Jr., LaMoreaux L, Werth JL, Poole RM. Clinical importance of changes in chronic pain intensity measured on an 11-point numerical pain rating scale. Pain. 2001;94:149-58.

3.3 REFERENCIAS DE PUBLICACIONES CIENTÍFICAS

Citar de forma completa (autor, título, publicación, fecha etc.) las referencias más relevantes utilizadas para el método (máximo 10 referencias)

1. Shahidi B, Maluf KS. Adaptations in Evoked Pain Sensitivity and Conditioned Pain Modulation after Development of Chronic Neck Pain. Biomed Res Int. 2017;2017:8985398.

2. Mlekusch S, Schliessbach J, Camara RJ, Arendt-Nielsen L, Juni P, Curatolo M. Do central hypersensitivity and altered pain modulation predict the course of chronic low back and neck pain? Clin J Pain. 2013 Aug;29(8):673-80.

3. Vaegter HB, Palsson TS, Graven-Nielsen T. Facilitated Pronociceptive Pain Mechanisms in Radiating Back Pain Compared With Localized Back Pain. J Pain. 2017 Aug;18(8):973-83.

4. Woolf CJ. Central sensitization: implications for the diagnosis and treatment of pain. Pain. 2011 Mar;152(3 Suppl):S2-15.

5. O'Leary H, Smart KM, Moloney NA, Blake C, Doody CM. Pain Sensitization Associated with Non-Response Following Physiotherapy in People with Knee Osteoarthritis. Pain. 2018 May 22.

6. Yarnitsky D, Granot M, Granovsky Y. Pain modulation profile and pain therapy: between pro- and antinociception. Pain. 2014 Apr;155(4):663-5.

7. Vaegter HB, Graven-Nielsen T. Pain modulatory phenotypes differentiate subgroups with different clinical and experimental pain sensitivity. Pain. 2016 Jul;157(7):1480-8.

8. Bossmann T, Brauner T, Horstmann T. Differences in pain intensity in anti- and pro-nociceptive pain profile subgroups in patients with knee osteoarthritis. Pain Management. 2018 01/01; 2018/07;8(1):27-36.

9. Petersen KK, Graven-Nielsen T, Simonsen O, Laursen MB, Arendt-Nielsen L. Preoperative pain mechanisms assessed by cuff algometry are associated with chronic postoperative pain relief after total knee replacement. Pain. 2016 Jul;157(7):1400-6.

10. Petersen KK, Arendt-Nielsen L, Simonsen O, Wilder-Smith O, Laursen MB. Presurgical assessment of temporal summation of pain predicts the development of chronic postoperative pain 12 months after total knee replacement. Pain. 2015 Jan;156(1):55-61.

3.4 INTERVENCIONES O PRUEBAS EN SERES HUMANOS

¿En esta actividad se realizan intervenciones o pruebas en seres humanos?

Sí

Entrevistas, encuestas, cuestionarios, etc.

Pruebas físicas

3.4.1 PROCESO DE RECLUTAMIENTO

Describir el proceso de reclutamiento: presentación del proyecto y obtención de consentimiento del sujeto fuente y/o su representante legal de acuerdo al siguiente esquema:

- quién (persona/s encargada/s)
- cómo (conversación, video, folleto informativo, etc),
- dónde (lugar de información y de firma de documentos)
- cuándo se realizan (momento en que se llevan a cabo)

El estudio se llevará a cabo en 2 locaclizaciones:

1- Clínica Hiru Fisioterapia SCP (Calle Antso Estegiz 4) :

Los sujetos que acudan a la clínica Hiru Fisioterapia solicitando tratamiento para una cervicalgia serán informados acerca de la realización de una investigación que estudia los procesamientos del dolor. Si desean más información al respecto, se les derivará a Josu Zabala Mata quien valorados los criterios de inclusión-exclusión, faciltará la hoja de consentimiento informado y el email de contacto. Puesto que la intervención descrita forma parte del tratamiento habitual del centro, los pacientes solamente tendrán una valoración más extensa de lo habitual, hecho del que se podrán beneficiar adquiriendo información para un diagnóstico más preciso.

2- Laboratorio del dolor de neurociencias de la UPV:

Un poster para sujetos control situado en la puerta del despacho de Josu Zabala Mata de la UPV anunciarán el estudio. Las personas que deseen más información podrán ponerse en contacto por email con Josu Zabala Mata, el cual procederá a enviarles la hoja de consentimiento informado.

Los criterios de inclusión para sujetos con dolor de cuello y sujetos control:

- Edad comprendida entre 20 y 60

Además, los sujetos con dolor de cuello presentarán:

- Dolor de cuello intermitente

- Que se agrava o mejora con determinados movimientos del cuello o determinadas posturas

- Que no haya sido causado por un golpe, caída o accidente

Criterios de exclusión para sujetos sintomáticos:

- Presenten una cervicobraquialgia

- Hayan sido o estén esperando una intervención quirúrgica del cuello

- Hayan sido diagnosticadas de una enfermedad inflamatoria o patología específica de la columna (por ejemplo artritis reumatoide)

- En cuyo tratamiento las movilizaciones cervicales y de los tejidos blandos estén contraindicadas

Criterios de exclusión para sujetos control:

- Presencia de dolor intermitente de más de 3 meses de duración en cualquier parte del cuerpo

- Patologías sistémicas, cardíacas o neurológicas que cursen con alteración de la sensibilidad

-Toma de medicación que altera la capacidad sensitiva

Fecha prevista de inicio del reclutamiento:

01/10/2018

3.4.2 RECLUTAMIENTO REALIZADO POR PERSONAL AJENO AL EQUIPO INVESTIGADO

¿El reclutamiento va a ser realizado por alguien ajeno al equipo investigador?

No

3.4.3 DESCRIPCIÓN DE LAS INTERVENCIONES O PRUEBAS

Describir todas las intervenciones o pruebas a las que se va a someter al sujeto de estudio para obtener sus muestras o sus datos especificando el lugar, persona encargada de realizarlas, duración y frecuencia.

Tras haber sido seleccionados para el estudio y aceptado el consentimiento informado, los sujetos pasarán a realizar las pruebas tal y como se detallará a continuación y en el orden descrito. Estas pruebas se repetirán una vez acabado el tratamiento y a un año vista. Dichas pruebas se realizarán en el centro Hiru Fisioterapia, ubicado en la localidad de Durango. El local estará acondicionado y controlado entre 21-22º de temperatura.

1. Variables descriptivas del sujeto. Tendrán que cumplimentar los cuestionarios con las siguientes preguntas:

- Edad, altura, peso

- Fecha de aparición de los síntomas

- Localización del dolor

- Intensidad media del dolor cervical en las últimas 24 horas, utilizando una escala visual analógica (Angmo et al. , 2016) y presencia de dolor durante los movimientos del cuello

- Discapacidad producida por la cervicalgia, utilizando el Índice de Discapacidad Cervical (Neck

Disability Index) validado al castellano (Andrade Ortega et al., 2010)

- Percepción catastrófica de la lesión, utilizando la escala de catastrofismo del dolor (Pain Catastrophizing Scale) validado al castellano (Campayo et al., 2008)

2. Variables e instrumentos de medición relativas a los parámetros del procesamiento del dolor

2.1. Umbral de dolor a la presión (PPT): las medidas de UDP se realizarán con un algómetro digital de presión FISHER con el punto de apoyo de 1 cm de diámetro (Pain Diagnostics and Thermography Inc, Great Neck, NY), tanto en el grupo sano como en el afecto. El UDP se define como la mínima presión con la que la sensación de presión se convierte en sensación dolorosa (Fisher et al., 1990). El UDP se aplicará en dos localizaciones diferentes y de forma bilateral. El primero será en el ángulo de las fibras superiores del trapecio, entre 5 y 8 cm superior y medial al ángulo superior de la escápula. El segundo punto será en el tibial anterior, 2.5 cm lateral y 5 cm inferior respecto a la tuberosidad tibial anterior. Los sujetos serán instruidos para que informen al investigador de la primera sensación de dolor percibida. La presión se realizará a un Kg por segundo hasta la señal del sujeto, momento donde se registrará el valor conseguido. Se aplicarán tres mediciones en cada localización, con un intervalo de 1 minuto, y se utilizará la media de los tres valores para el estudio. Este procedimiento mostró un alto coeficiente de correlación intraclase (Chesterton et al., 2007).

2.2. Sumación temporal del dolor (TSP)

2.2.1. Búsqueda del umbral del dolor: Las mediciones se realizarán en un ambiente tranquilo, con los sujetos sentados enfrente de una mesa donde apoyarán su mano. Dos electrodos autoadhesivos se colocarán en el dorso de la mano dominante, a dos cm de separación una de la otra. Se aplicará un tren de 5 impulsos con 1 ms de duración y 4ms de diferencia. En total 21 ms. Para ello utilizaremos un electroestimulador DS7, la cual tras calcular la impedancia de la piel donde se aplican los impulsos, modifica la potencia para que la intensidad de estímulo que le llega al individuo sea la mA deseados. El electroestimulador estará a su vez conectado al ordenador, el cual mediante un programa específico, posibilitará la aplicación de la frecuencia deseada. Se aplicará el tren de forma escalonada, subiendo la intensidad hasta conseguir el umbral del dolor. Dicha subida se realizará de 0.2 en 0.2 mA. A continuación, empezando por un mA por encima del umbral delimitado en la subida, iremos bajando a la misma frecuencia hasta que el estímulo se note como no doloroso. Este procedimiento se realizará dos veces y cogeremos la media de los 4 valores obtenidos.

2.2.2. Búsqueda del Wind Up: Protocolo; Aplicación de un tren de 5 trenes (5 impulsos en cada tren) con una frecuencia de 3 Hz y la intensidad de 1.2 respecto umbral. Primero se evaluará el dolor del primer impulso del primer tren mediante la escala verbal numérica de clasificación del dolor. Tras 3 minutos de descanso, se volverá a aplicar el mismo tren y se le pedirá que cuantifique la intensidad de dolor del último impulso. Por último sacaremos un ratio del aumento del dolor.

Este procedimiento no supone ningún sufrimiento para los sujetos ya que las aplicaciones del estímulo eléctrico están muy cerca del umbral del dolor.

2.3. Modulación condicionada al dolor (CPM): Realizaremos una medición del PPT con el algómetro digital (anteriormente citado) en el primer espacio intermetatarsal de la mano homolateral al dolor de cuello, o en caso de dolor bilateral, en la mano dominante. A continuación introduciremos el pie contralateral a la mano utilizada en un cubo de agua con hielos. Mantendremos el pie inmerso durante 2 minutos. En el caso que el sujeto no tolerase dicho tiempo se retirará el pie del agua. Volveremos a realizar las mediciones del umbral del dolor mediante algómetro y corriente eléctrica tanto durante (pasados 30 segundos) como inmediatamente después de la inmersión del pie en el agua. Utilizaremos los valores obtenidos de los umbrales del dolor antes, durante y después para medir el efecto de las vías inhibitorias del dolor. Esta metodología ha mostrado buena a excelente fiabilidad intrasesión (Lewis et al., 2012; Kennedy et al., 2016) [(PPTcold − PPTcontrol)/PPTcontrol] ∗ 100

3. Variables de medición relativas a los efectos de las intervenciones

3.1. Escala de impresión de mejoría global del paciente (PGI-I: Patient Global Impression of Improvement Scale): Se utilizará el PGI-I para medir la respuesta sintomática del participante a la intervención (Farrar et al., 2001). El PGI-I consiste en una sola pregunta que solicita al participante que clasifique el alivio obtenido con la intervención. El participante escoge de una escala likert de 7 puntos (Muchísimo mejor; Mucho mejor; Un poco mejor; Ningún cambio; Un poco peor; Mucho peor; Muchísimo peor) la respuesta que más se ajuste al efecto. Se utilizará la version española desarrollada por la Sociedad Española del Dolor.

4. Personas que realizarán la intervención y mediciones:

Todas las mediciones se realizarán por la misma persona (Josu Zabala Mata) para asegurar

una adecuada estandarización de las mismas. Las mediciones tendrán una duración aproximada de 1 hora. El estudio se realizará en la consulta Hiru Fisioterapia SCP, del Fisioterapeuta Josu Zabala Mata. Las intervenciones las realizarán los fisioterapeutas Araitz Irazu Legorburu y Mikel Larrañaga Etxaburu.

5. Intervención (sólo para sujetos con dolor de cuello)

Se realizará un tratamiento de fisioterapia con una duración máxima de 4 semanas, aplicando una intervención semanal de alrededor de 45 minutos. El tratamiento consistirá en tres vías de trabajo; por un lado se trabajará el componente miofascial, por otro el componente articular y por último el control motor. Para el componente miofascial se realizarán técnicas fasciales y tratamiento de los puntos gatillos miofasciales (PGM). El tratamiento articular consistirá en movimientos pasivos a baja velocidad, incluyendo translaciones segmentarios, deslizamientos accesorios y movimientos fisiológicos tanto durante como al final del recorrido articular. El control motor se trabajará en consulta y se mandarán ejercicios específicos para casa. La realización de las diferentes técnicas dependerá de la valoración fisioterapéutica realizada el primer día y de la evolución del tratamiento. Dicha valoración será realizada por el investigador principal y el tratamiento lo realizarán 2 fisioterapeutas con una experiencia en terapia manual de más de 10 años.

El tratamiento propuesto es el que se realiza de forma habitual en este centro de fisioterapia, con lo que no supone ningún cambio para los pacientes.

3.4.4 INCENTIVOS PARA LA PARTICIPACIÓN

¿Se va dar algún tipo de incentivo o compensación al sujeto de estudio por su participación?

No

3.5 MUESTRAS BIOLÓGICAS DE ORIGEN HUMANO

¿En esta actividad se utilizan muestras biológicas de origen humano?

No

3.5.1 ORIGEN DE LAS MUESTRAS

¿Cómo se han obtenido las muestras?

3.5.2 DESTINO DE LAS MUESTRAS DE ORIGEN HUMANO

¿Qué va a hacer con las muestras de origen humano una vez que ya no sean necesarias para este proyecto?

3.6 DATOS DE CARÁCTER PERSONAL

¿En esta actividad se utilizan datos de carácter personal?

Sí

Nº de registro del tratamiento de datos RGPD

[redacted]

Parte IV: Aspectos éticos específicos

4.1 PONDERACIÓN DE LOS RIESGOS O MOLESTIAS

Completar los siguientes apartados sólo si se ha seleccionado “Sí” en el apartado "3.4 Intervenciones o pruebas en seres humanos"

4.1.1 TIPO DE RIESGOS O MOLESTIAS

Señale a continuación el tipo de riesgos y/o molestias que se ocasionarán al sujeto de investigación debido a las intervenciones o pruebas que se le realizarán para el proyecto.

Riesgo físico

Uso de su tiempo

4.1.2 VALORACIÓN DE LOS RIESGOS O MOLESTIAS

Teniendo en cuenta las intervenciones y pruebas que se van a realizar a los sujetos de investigación descritas en el apartado 3.4.3 ¿cómo valoraría los riesgos o molestias posibles según la siguiente definición?

Riesgo mínimo: aquel que no supera en probabilidad o magnitud al que cabría esperar en la actividad cotidiana, incluyendo exámenes físicos o psicológicos rutinarios

4.1.3 MINIMIZACIÓN DEL RIESGO O MOLESTIAS SUPERIORES AL MÍNIMO

¿Qué mecanismos se han previsto para reducir el riesgo o las molestias que se han calificado como superiores al mínimo?

4.1.4 ASEGURAMIENTO DEL DAÑO

¿Se dispone de un seguro de responsabilidad civil (seguro universitario, escolar u otros) que de cobertura en todos los aspectos del proyecto de investigación?

No

Sí

Nº de póliza

[redacted]

4.2 SELECCIÓN EQUITATIVA DE LA MUESTRA

Justificar la elección de la muestra de estudio y describir las medidas que garantizan la equidad en el proceso de reclutamiento.

La selección de los sujetos se hará en base a lo descrito en la sección 3.4.1 y solo se aplicarán los criterios descritos en esa sección, sin discriminar a los sujetos por ningún otro criterio.

4.3 PROTECCIÓN DE GRUPOS VULNERABLES

¿Se trata de personas vulnerables? (menores, personas legalmente incapacitadas etc.)

No

4.4 INFORMACIÓN Y CONSENTIMIENTO

¿Se solicita el consentimiento de la persona participante/fuente y/o su representante legal?

Sí

4.4.1 TIPO DE CONSENTIMIENTO

Tarea para la que se solicita el consentimiento

Para realizar a la persona participante intervenciones no invasivas

Para uso de las muestras y/o los datos de la persona participante en el proyecto actual

En los siguientes apartados se hace un repaso a la información que va a facilitarse al sujeto participante y puede servir de lista de chequeo para el investigador responsable.

4.4.2 INFORMACIÓN OBLIGATORIA MÍNIMA

El documento de consentimiento debe llevar los logos UPV/EHU y de otras instituciones si procede.

Los datos identificativos y de contacto deben coincidir con los aportados previamente en esta memoria.

A continuación, se le facilita la información que debe incluir en el documento de consentimiento para su proyecto de investigación

4.5 ESTUDIOS GENÉTICOS

¿Se va a realizar algún tipo de prueba predictiva genética?

No

4.6 ESTUDIANTES COMO SUJETOS DE INVESTIGACIÓN

¿Participan estudiantes de la UPV/EHU como sujetos de investigación?

No

Parte V: Cumplimiento de documentación

5.1 DOCUMENTO DE CONSENTIMIENTO

¿Su proyecto requiere documento de consentimiento del sujeto fuente y/o su representante?

Sí

Documentos asociados a la pregunta:

Grupo intervención

Grupo control

5.2 DOCUMENTO DE CESIÓN DE DATOS

¿Su proyecto requiere la autorización de la Agencia Española de Protección de Datos (AEPD) para cesión de datos?

Sí

5.3 DOCUMENTO DE AUTORIZACIÓN

Si parte de su proyecto se realiza fuera de las instalaciones de la UPV/EHU ¿dispone Documento de Autorización del Responsable del Centro o lugar?

Sí

5.4 DOCUMENTO DE ACEPTACIÓN DE COLABORACIÓN

Si el reclutamiento de las personas participantes va a ser realizado por alguien ajeno al equipo investigador ¿dispone del documento de aceptación de colaboración?

Sí

5.5 INFORME DE LA COMISIÓN DE GARANTÍAS

¿Su proyecto requiere informe previo favorable de la Comisión de Garantías?

No

5.6 ACUERDO DE TRASFERENCIA DE MUESTRAS

Si su proyecto ha requerido Cesión de Muestras o Tejidos ¿dispone de un Acuerdo de Transferencia de Muestras o Tejidos Humanos (MTA)?

No

5.7. DOCUMENTO DE REGISTRO DE TRATAMIENTO DE DATOS RGPD

¿Su proyecto requiere la autorización mediante el documento de resgistro de tratamiento de datos RGPD?

Sí

5.8 DOCUMENTACIÓN ADICIONAL

¿Dispone de alguna otra documentación adicional para su proyecto?

Sí

Documentos asociados a la pregunta:

Seguro civil individ

Seguro civil centro

Poster

5.9 ACLARACIONES AL COMITÉ

Incluir a continuación cualquier otra aclaración que se considere necesaria para el comité

5.4. Existen dos personas que colaboran en el proyecto y adjunto el documento pertinente, pero no participan en el reclutamiento, sino que el la intervención.

5.7. Aunque tenga hecho todo el procedimiento para el registro de tratamiento de datos RGPD y posea un número de referencia del fichero, acaba de haber un cambio en la ley que obliga a modificar el formato del archivo a adjuntar. Dado que el responsable de este tema es Andoni Juaristi y actualmente está de vacaciones, no podré adjuntar este documento y realizaré dicho apartado una vez recibido la respuesta del comité de ética.
