# Supplementary material for: Restoration of normal central pain processing following manual therapy in nonspecific chronic neck pain
Source: PLoS One. 2024 May 23;19(5):e0294100. doi: 10.1371/journal.pone.0294100 (PMC11115211; doi:10.1371/journal.pone.0294100)
Supplement: Attachment — Submitted filename: Reviewers comments for neck pain study.docx [file pone.0294100.s005.docx]

Reviewer's Report:

Title: Restoration of normal central pain processing following manual therapy in nonspecific chronic neck pain.

Abstract:

1. Lack of Specific Results: The abstract mentions that "an increased CPM response and attenuated TSP were found," but it does not provide specific quantitative results or effect sizes, making it difficult for readers to assess the significance of these findings.

2. Minor Associations: The abstract states that "only minor associations were found between normalization of TSP/CPM and measures of clinical outcome," which raises questions about the clinical significance of the observed changes in central pain processing.

3. Limited Information on Participants: It would be helpful to include some basic demographic information about the participants in the abstract to provide context for the study findings.

**Introduction:**

The introduction section provides valuable background information on the prevalence and challenges associated with non-specific chronic neck pain (NSCNP). However, there are some points to consider:

1. Lack of Clarity on Research Gap: While the introduction highlights the need for better understanding the mechanisms of manual therapy, it does not clearly specify the research gap or the specific questions the study aims to address.

2. Reference to Guidelines: The introduction mentions guidelines advocating exercise and manual therapy for NSCNP but does not provide specific references or citations, which could enhance the credibility of the claims.

3. Lengthy Background Information: The introduction contains extensive background information on the prevalence of neck pain, which, while informative, could be condensed for brevity.

**Methods:**

1. Sample Size Justification: The rationale for the sample size of 63 participants is based on the detection of a maximum difference of 10% in TSP and CPM measures, but it would be beneficial to provide more information on how this specific effect size was determined.

2. Lack of Control Group: The study lacks a control group, which makes it challenging to establish causation and attribute the observed changes solely to the manual therapy intervention.

3. Treatment Description: The methods describe the manual therapy intervention broadly but do not provide specific details about the techniques used or the treatment protocol, which limits the ability to replicate the study.

4. Statistical Analysis: While the statistical approach is mentioned, the methods could benefit from a more detailed explanation of the statistical models used and the rationale for choosing the Bayesian approach.

**Results and Discussion**

1. Lack of Control Group: One of the major limitations of this study is the absence of a control group. Without a control group, it is challenging to attribute the observed improvements in central pain processing and clinical outcomes solely to the manual therapy intervention. It is essential to account for the natural course of the condition and any potential placebo effects.

2. Weak Associations: The study reports very weak associations between changes in central pain processing mechanisms and clinical/psychological variables. This suggests that other factors or mechanisms might be at play in explaining the clinical improvement following manual therapy. The authors acknowledge this limitation but do not provide a more in-depth discussion on possible alternative explanations or mechanisms.

3. Methodological Differences: The authors mention methodological differences in stimulus types used in previous studies and the current study for evaluating central pain processing (TSP). However, the significance and potential implications of these differences are not discussed in detail in the Discussion section.

4. Implications for Clinical Practice: The manuscript could benefit from a more comprehensive discussion of the clinical implications of the findings. How can these results inform clinical practice, and what recommendations can be made for manual therapy in the treatment of NSCNP patients?

5. Sample Characteristics: The manuscript briefly mentions that the sample presented only mild baseline disability. This should be discussed more thoroughly, as it may have implications for the generalizability of the findings to the broader NSCNP population.

6. Limitations: The limitations section is somewhat brief. It would be beneficial to provide a more extensive discussion of the study's limitations, including the potential impact of the lack of a control group and the generalizability of the findings.

Conclusion: Authors did not written conclusion
