# Supplementary material for: Restoration of normal central pain processing following manual therapy in nonspecific chronic neck pain
Source: PLoS One. 2024 May 23;19(5):e0294100. doi: 10.1371/journal.pone.0294100 (PMC11115211; doi:10.1371/journal.pone.0294100)
Supplement: Attachment — Submitted filename: Review comments for- NSCNP- manual therapy-PLOSOne.docx [file pone.0294100.s006.docx]

I am greatly happy to review this article**-** Manuscript Number: **PONE-D-23-34005R1 - Restoration of normal central pain processing following manual therapy in nonspecific chronic neck pain. Manuscript is written very well, methodology need more detail, study findings are very interesting** There are a few points that need to be addressed. The study is good and very helpful for clinical Physical Therapy Health Professionals.

1- **Abstract: Revised abstract sound good.**
2- **What was reason of high percentage of female participants with NSCNP, is there any reference supporting high prevalence of pain in female.**
3- **Methodology: Need more detailed procedure for manual therapy, like who did this procedure of mobilization, is it the same researcher, who evaluated pre-and post intervention outcomes or other, was he certified manual therapist with how many years of experience?**
4- **P-A mobilization with how many, thrust or oscillations in each direction.**
5- **Mobilization force was applied at spinous process or at transvers process, if so how did you identified or located these landmarks.**
6- **While giving mobilization force vertebra was stabilized for example C4-C5 vertebra there is hypomobility.**
7- **How did authors gave A-P mobilizations at cervical spine.**
8- **Please attach few pictures of manual therapy, placement of hands, direction of mobilization etc..**
9- **How did authors calculated sample size (63), please write formula.**
10- **How reliable and valid was the Questionnaire used in this study?**
11- **Findings are very interesting.**

**Thanks & Regards.**
